# Dietary and Pharmacologic Manipulations of Host Lipids and Their Interaction With the Gut Microbiome in Non-human Primates

**DOI:** 10.3389/fmed.2021.646710

**Published:** 2021-08-26

**Authors:** Jennifer M. Lang, Leslie R. Sedgeman, Lei Cai, Joseph D. Layne, Zhen Wang, Calvin Pan, Richard Lee, Ryan E. Temel, Aldons J. Lusis

**Affiliations:** ^1^Departments of Medicine, Microbiology and Human Genetics, University of California, Los Angeles, Los Angeles, CA, United States; ^2^Department of Medicine, Division of Cardiology, University of California, Los Angeles, Los Angeles, CA, United States; ^3^Cardiovascular Research Center, University of Kentucky, Lexington, KY, United States; ^4^Cardiovascular and Metabolic Diseases, Novartis Institutes for Biomedical Research, Cambridge, MA, United States; ^5^Department of Physiology, School of Basic Medical Sciences, Shandong University, Jinan, China; ^6^Ionis Pharmaceuticals, Carlsbad, CA, United States; ^7^Department of Physiology, University of Kentucky, Lexington, KY, United States

**Keywords:** microbiome, primate, lipids, high-fat diet, bile acids

## Abstract

The gut microbiome influences nutrient processing as well as host physiology. Plasma lipid levels have been associated with the microbiome, although the underlying mechanisms are largely unknown, and the effects of dietary lipids on the gut microbiome in humans are not well-studied. We used a compilation of four studies utilizing non-human primates (*Chlorocebus aethiops* and *Macaca fascicularis*) with treatments that manipulated plasma lipid levels using dietary and pharmacological techniques, and characterized the microbiome using 16S rDNA. High-fat diets significantly reduced alpha diversity (Shannon) and the Firmicutes/Bacteroidetes ratio compared to chow diets, even when the diets had different compositions and were applied in different orders. When analyzed for differential abundance using DESeq2, *Bulleidia, Clostridium, Ruminococcus, Eubacterium, Coprocacillus, Lachnospira, Blautia, Coprococcus*, and *Oscillospira* were greater in both chow diets while *Succinivibrio, Collinsella, Streptococcus*, and *Lactococcus* were greater in both high-fat diets (oleic blend or lard fat source). Dietary cholesterol levels did not affect the microbiome and neither did alterations of plasma lipid levels through treatments of miR-33 antisense oligonucleotide (anti-miR-33), Niemann–Pick C1-Like 1 (NPC1L1) antisense oligonucleotide (ASO), and inducible degrader of LDLR (IDOL) ASO. However, a liver X receptor (LXR) agonist shifted the microbiome and decreased bile acid levels. Fifteen genera increased with the LXR agonist, while seven genera decreased. *Pseudomonas* increased on the LXR agonist and was negatively correlated to deoxycholic acid, cholic acid, and total bile acids while *Ruminococcus* was positively correlated with taurolithocholic acid and taurodeoxycholic acid. Seven of the nine bile acids identified in the feces significantly decreased due to the LXR agonist, and total bile acids (nmol/g) was reduced by 62%. These results indicate that plasma lipid levels have, at most, a modest effect on the microbiome, whereas bile acids, derived in part from plasma lipids, are likely responsible for the indirect relationship between lipid levels and the microbiome.

## Introduction

The microbiome plays an integral role in host dynamics of health and disease, and this relationship is tied to diet. Decreased health status has been associated with microbiome dysbiosis (1–4), high-fat diets (5, 6), and increased plasma lipid levels (7). Cholesterol and its lipoprotein carriers are important contributors to cardiovascular disease, and recent evidence suggests that the microbiome may play a role in this relationship by influencing lipid metabolism (8–11).

Lipid levels in the blood are used as an indicator of health, with about 40% of lipid variation attributed to genetics (12). The rest is explained by the environment, which would include diet and, more recently, the microbiome. Lipids have been correlated to microbial taxa in mice (8), pigs (13), and humans (14, 15), but the identified taxa have varied across the studies. One recurring result is that increased alpha diversity is correlated to elevated high-density lipoprotein (HDL) and reduced triglyceride levels, but low-density lipoprotein (LDL) appears to have little or no association with the microbiome (14–16). Plasma cholesterol was influenced by manipulations of the microbiome through antibiotic reduction and fecal transplants and demonstrated a causal relationship between the microbiome biome and circulating cholesterol (17).

Dietary lipids have a strong influence on host lipid levels as well as the microbiome. High-fat diets are considered unfavorable to health because they are related to increased LDL levels (18) and cardiovascular disease, as well as “starving” the microbiome by reducing microbially accessible carbohydrates and fermentable fibers (19, 20). It is important to note that fat composition (monounsaturated vs. polyunsaturated vs. saturated) (21, 22), source (lard vs. fish oil) (23), and quality (oxidized vs. unoxidized) are all characteristics that influence the effect of fat.

Lipid metabolism and cholesterol levels are influenced by bile acids, which are cholesterol derivatives synthesized in the liver and secreted in bile via the gall bladder into the small intestine (24). In the intestine, bile acids modulate bacterial composition by restricting bacterial proliferation and growth (25). In turn, bacterial enzymes modify bile acids through deconjugation, epimerization, and dehydroxylation to produce secondary bile acids (26). Ninety-five percent of bile acids in the intestine are then reabsorbed into the enterohepatic circulation for further modification, whereas 5% are excreted in feces. Germ-free rats have been shown to have a reduction in circulating bile acids (27), especially a reduction in secondary bile acids, compared to conventionally raised rats. Furthermore, the role of bile acids in signaling through the farnesoid-X-receptor (FXR) and the G-protein coupled receptor, TGR5, may represent an additional mechanism through which the microbiome interacts with the mammalian host (28, 29).

It has proven difficult to determine the causal interactions between the gut microbiome and plasma lipids directly in humans. We have therefore attempted to address this issue using studies of non-human primates. Here we use a compilation of four non-human primate (NHP) studies with dietary and pharmacological treatments that perturbed host lipid levels. We found that high-fat diets with different fat compositions and opposite ordering of diet treatments had similar effects on the microbiome, but that the microbiome did not significantly respond to changes in dietary cholesterol and two pharmacological treatments that perturbed plasma lipid metabolism. A pharmacological treatment that did affect the microbiome was a liver X receptor (LXR) agonist, which decreased levels of bile acids. These results indicate that plasma lipid levels do not, or minimally, affect the microbiome but that bile acids may be responsible for creating an indirect relationship between the microbiome and plasma lipid levels.

## Materials and Methods

### High-Fat Diet Oleic Blend Study Design

The design of this study was previously described (30) as follows: Adult male African green monkeys (*Chlorocebus aethiops*) (*n* = 15, age 5–10 years) were obtained from St. Kitts Island. Monkeys were housed in an Assessment and Accreditation of Laboratory Animal Care (AAALAC)-accredited facility under the direct care of the Wake Forest School of Medicine Animal Resources Program and euthanized at the termination of the study. All experiments were approved by the Institutional Animal Care and Use Committees of Wake Forest School of Medicine. Monkeys were singly housed in climate-controlled conditions with a 12 h light and dark cycle. The monkeys were provided water *ad libitum* and were initially fed a weighed amount of a chow diet (Monkey Diet 5038, Lab Diet) twice daily, such that their daily caloric intake was 70 kcal day/kg body weight. During the 10-week experimental diet feeding phase, the monkeys were fed twice daily with a weighed amount of semi-synthetic diet containing 0.002 (Lo), 0.2 (Med), or 0.4 (Hi) mg cholesterol/Kcal, which was prepared at the Wake Forest Primate Center Diet Laboratory. Daily caloric intake was 90 kcal/day/kg body weight. Fecal samples were collected before and at the completion of the 10-week experimental diet feeding and stored at −20°C until extraction.

### Recovery From High-Fat Diet Study Design

Young adult male cynomolgus monkeys (*Macaca fascicularis*) (*n* = 73) originated from Mauritius and at the onset of the study were an average age of 5.3 years (range 4.2–6.7). The monkeys were housed in an AAALAC-accredited facility under the direct care of the University of Kentucky (UK) Division of Laboratory Animal Resources (DLAR), and all experiments were approved by the UK Institutional Animal Care and Use Committee. Monkeys were housed in climate-controlled conditions with a 12 h light and dark cycle and initially fed *ad libitum* a standard non-human primate chow diet (Teklad 2050). The monkeys were then singly housed from ~08:00–15:00 each day and in the morning and afternoon, received weighed portions of a semi-synthetic high-fat atherogenic diet (Table 1), which provided on average 74 kcal/kg body weight/day. After 20 months on the high-fat diet, monkeys were switched back to standard chow diet and were treated for 6 weeks or 6 months with either vehicle (USP grade saline) or miR-33a/b antagonist RG428651, a 2′-fluoro/methoxyethyl-modified, phosphorothioate (PS)-backbone-modified, antisense oligonucleotide (ASO) (Regulus Therapeutics). Monkeys were injected subcutaneously with vehicle or 5 mg ASO/kg body weight twice weekly during the first 2 weeks and then once weekly during the remainder of the study. During the treatment period, animals were singly housed from ~08:00–15:00 each day and received 12 biscuits of standard diet, which provided on average 64 kcal/kg body weight/day. Cecum samples were collected from monkeys at completion of the 20-month high-fat diet and then from monkeys that were switched to chow for 6 weeks or 6 months post high-fat diet.

**Table 1:** Macronutrient composition as percent total calories.

	**High-Fat Diets**				**Chow**	
	**Oleic**	**High**	**Medium**	**Low**	**Oleic Blend**	**Recovery Study**
	**Blend**	**Cholesterol**	**Cholesterol**	**Cholesterol**	**Study (5038)**	**(2050)**
Carbohydrates (%)	46	46	46	46	69	57
Protein (%)	17	16	16	17	18	29
Lipid (%)	37	38	38	37	13	14
Saturated (% lipid)	23	46	46	46	38	23
Monounsaturated (% lipid)	64	40	40	40	48	31
Polyunsaturated (% lipid)	13	19	19	19	14	46

### Biliary Cholesterol Study Design

Male cynomolgus monkeys (*Macaca fascicularis*) (*n* = 12) originated from China and at the onset of the study were an average age of 3.6 years (range 3.8–3.4). The monkeys were housed in an AAALAC-accredited facility under the direct care of the University of Kentucky (UK) Division of Laboratory Animal Resources (DLAR), and all experiments were approved by the UK Institutional Animal Care and Use Committee. Monkeys were housed in climate-controlled conditions with a 12 hr light and dark cycle. The NHPs were fed a standard non-human primate chow diet (Teklad 2050) for the first 4 weeks of treatment and then switched to a high-fat/low cholesterol diet for the last 4 weeks of treatment. They were then singly housed from ~08:00–15:00 each day and in the morning and afternoon, received either 6 standard diet biscuits/feeding or weighed portions of the high-fat/low cholesterol diet, which provided ~100 kcal/kg body weight/day. Animals were injected subcutaneously twice a week for 8 weeks with control antisense oligonucleotide (ASO) (*n* = 6; Ionis 141923) or Niemann-Pick C1-Like 1 (NPC1L1) ASO (*n* = 6; Ionis 807400) at 20 mg/kg/dose. Feces were collected for 4 consecutive days during the 8th week of treatment and stored at −20°C until extraction.

### Quantification of NPC1L1 mRNA

Total RNA was isolated from frozen liver and jejunum using RNAzol Reagent (Molecular Research Center, RN190) according to the manufacturer's instructions. The concentration of the RNA was determined using an ND-1000 UV-Visible Spectrophotometer (Nanodrop). The integrity of the RNA was verified by evaluating ribosomal RNA bands (28S/18S) separated on a 1% denaturing agarose gel. Complementary DNA was synthesized from 2 μg of total RNA using High-Capacity cDNA Reverse Transcription Kit with RNase Inhibitor (Applied Biosystems, 4374966). The cDNA was subjected to quantitative real-time PCR reaction using TaqMan™ Fast Advanced Master Mix (Applied Biosystem, 4444557) and TaqMan Gene Expression Assay containing a set of PCR primers and a TaqMan probe for cynomolgus monkey NPC1L1 and PAK1IP1 (Applied Biosystem; 4351372, Assay ID: NPC1L1, Mf02793772_m1; PAK1IP1, Mf02855023_m1). PCR was performed on Biorad C1000 Touch Thermal Cycler with the following conditions: 95°C for 30 s, followed by 40 cycles at 95°C for 10 s and 57°C for 25 s. The relative amount of target gene was normalized to the amount of PAK1IP1 (internal control) using the 2-ΔΔCT method.

### Lipid Homeostasis Study Design

The design of this study was previously described (31) as follows: All experiments were approved by the WFUHS Institutional Animal Care and Use Committee (IACUC). Male cynomolgus monkeys (*n* = 16) were obtained from the Wake Forest University breeding colony and were an average age of 2.0 years (range 1.8–2.6). Animals were pair-housed when possible in climate-controlled conditions with 12 h light/dark cycles. For the combined LXR agonist (GW3965) and inducible degrader of LDLR (IDOL) ASO treatment study, 16 monkeys were fed a high-fat/moderate cholesterol diet (Teklad) for 4 weeks and subcutaneously injected once during study week 1 with vehicle (USP grade saline) or 40 mg/kg IDOL ASO (Ionis 549127; Ionis Pharmaceuticals) and once during study weeks 2–9 with vehicle or 30 mg/kg IDOL ASO. Starting on study week 8, monkeys were fasted overnight, anesthetized as above, and gavaged with 10 mg/kg GW3965. The treatment regimen was conducted once daily for 8 days. Feces were collected on 3 consecutive days during study weeks 7 and 8 of ASO treatment.

### Plasma Lipid and Lipoprotein Concentration

For all previously mentioned studies, monkeys were sedated following an overnight fast with ketamine (10 mg/kg intramuscularly), and blood was collected in EDTA-containing Vacutainer® tubes. Plasma was isolated by centrifugation at 1,500 × g for 30 min at 4°C. Total plasma cholesterol (Pointe Scientific, C7510) and triglyceride (Sigma, TR0100 & F6428) concentrations were measured using enzymatic kits. The cholesterol distribution among lipoprotein classes was determined after separation by gel filtration chromatography based upon the method described previously (32). An aliquot of plasma from each animal was diluted to 0.5 μg total cholesterol/μL in 0.9% NaCl, 0.05% EDTA/NaN_3_ and centrifuged at 2,000 × g for 10 min to remove any particulate debris. The supernatant was transferred to a glass insert contained in a GC vial. After loading the vial into an autosampler set at 4°C (Agilent Technologies, G1329A), 40 μL of sample was injected onto a Superose 6 10/300 (GE Healthcare Life Sciences) chromatography column. Under the control of an isocratic pump (Agilent Technologies, G1310A/B), the sample was separated at a flow rate of 0.4 ml/min with eluent containing 0.9% NaCl, 0.05% EDTA/NaN_3_. The column effluent was mixed with cholesterol reagent (Pointe Scientific, C7510) running at a flow rate of 0.125 mL/min and the mixture was passed through a knitted reaction coil (Aura Industries Inc., EPOCOD) in a 37°C water jacket. The absorbance of the reaction mixture was read at 500 nm using a variable wavelength detector (Agilent Technologies, G1314F). The signal was subsequently integrated using Agilent OpenLAB Software Suite (Agilent Technologies). VLDL-C, LDL-C, and HDL-C concentrations were determined by multiplying the plasma total cholesterol concentration by the cholesterol percentage within the elution region for each lipoprotein class.

### Analysis of Hepatic Cholesterol

Frozen liver (~50 mg) was thawed, minced, and weighed in a 16 × 100 mm glass screw-top tube with a Teflon lined cap. Lipids were extracted in 3 ml 2:1 chloroform/methanol at 60°C for 3 h and then at room temperature overnight. The lipid extract plus a 2 ml 2:1 chloroform/methanol wash of the extraction tube was transferred to a new glass tube and dried down under N_2_ at 60°C. After the lipid was resuspended in 6 ml 2:1 chloroform/methanol, 1.2 ml 0.05% sulfuric acid was added, the tube was vortexed, and the phases split by centrifugation at 1,500 × g at room temperature for 10 min. The same volumes of 2:1 chloroform/methanol and 0.05% sulfuric acid were added to an empty 15 ml graduated glass tube to determine the volume of the bottom phase. The aqueous upper phase was aspirated and discarded, and an aliquot (typically 1 ml) of the bottom phase was transferred to a new glass tube using a glass volumetric pipet. To the aliquot of the bottom phase was added 2 ml 1% Triton X-100 in chloroform, and the solvent was evaporated as described above. Deionized water (1 ml) was then added to the tube which was capped, heated at 60°C for 10 min, and periodically vortexed until the solution was clear. The samples and cholesterol standards containing 2% Triton-X100 were analyzed for total cholesterol (Pointe Scientific, C7510) and free cholesterol (Wako, 993-02501) using enzymatic assays conducted in 96-well plates.

### Measurement of Cholesterol in Bile

Gall bladder bile (10 μl) was transferred to a 16 × 100 mm glass screw top tube with a Teflon lined cap containing 0.75 ml ddwater and 10 μg 5-alpha cholestane (Steraloids, C3300-000). The following solutions were sequentially added to the tube with vortexing for 20 s after each addition: (1) 2.25 ml 2:1 methanol:chloroform, (2) 1.5 ml chloroform, (3) 0.75 ml ddwater. The tube was centrifuged at 1,500 × g at room temperature for 10 min. After removing the top phase, a portion of the bottom phase was transferred to a new glass tube and evaporated under N2 at 60°C. Ninety five percentage ethanol (1 ml) and 1 ml 50% KOH was added and the lipid was saponified by incubating the tube at 60°C for at least 60 min. The following solutions were sequentially added to the tube with vortexing for 20 s after each addition: (1) 1 ml hexane, (2) 1 ml ddwater. The tube was centrifuged at 1,500 × g at room temperature for 10 min. The upper hexane phase was transferred to a 12 × 75 mm glass tube and evaporated under N2 at 60°C. The dried lipid was dissolved in 50–100 μl hexane and transferred to a teardrop GC vial insert. The neutral sterols were analyzed by injecting 1 μL of sample onto a ZB50 (0.53-mm inner diameter × 15 m × 1 μm) gas-liquid chromatography column (Phenomenex) at 250°C and installed in an Agilent Technologies 7890B gas chromatograph equipped with a Agilent Technologies 7,693 autosampler using on-column injection and a flame ionization detector.

### Extraction of Fecal Bile Acids

Fecal bile acids were extracted as previously described with minor modifications (33). Monkey fecal samples were weighed (150–300 mg each) in a glass tube. HPLC, or LC-MS grade solvents were used throughout the extraction procedure (FisherScientific). Samples were homogenized in 1 ml of ethanol containing 0.1N sodium hydroxide (Fisher Scientific) using an Omni Tissue homogenizer (Omni International). Samples were brought up to 5 ml in ethanol and 0.5 nmol of internal standard glycocholic acid (GCA; Sigma-Aldrich) was added to each sample. Samples were placed in an 80°C water bath for 1 h and then cooled on ice. Samples were centrifuged at 1,500 × g for 10 min and supernatants were dried using an EZ-2 Evaporator (Genevac). Dried samples were resuspended in HPLC-grade water and then subjected to a solid-phase extraction using a HyperSep-C18 cartridge (ThermoFisher). C18 cartridges were preconditioned with methanol:chloroform mixture (2:1), methanol and water. After loading the samples, cartridges were washed with water, n-hexanes, and allowed to dry for 5 min. Samples were eluted in 1 mL of methanol. Internal standard recovery ranged from 70 to 90% and was used to calculate extraction efficiency.

### Fecal Neutral Sterol Analysis

After a 3–4 day quantitative collection of feces from singly housed animals, water was added to the feces and total weight of the mixture was determined. After making a fecal slurry using a blender, an aliquot (~100 μl) was transferred to a 16 × 100 mm glass screw top tube containing 100 μg 5-alpha cholestane (Steraloids, C3300-000). After weighing the tube to determine the exact weight of the fecal slurry aliquot, 2 ml 95% ethanol and 200 μl 50% KOH was added. The tube was sealed with a Teflon lined cap and incubated at 60°C for 3 h with periodic vortexing. The following solutions were sequentially added to the tube with vortexing for 20 s after each addition: (1) 2 ml hexane, (2) 2 ml ddwater. The tube was centrifuged at 1,500 × g at room temperature for 10 min. The upper hexane phase was transferred to a glass GC vial and analyzed for coprostanol and cholesterol levels with GC-FID as described elsewhere in the manuscript for biliary cholesterol.

### LC-MS/MS

Identification and quantification of bile acids was achieved by LC-MS using an Ultimate 3,000 UHPLC liquid chromatography system (ThermoScientific) equipped with a C18-PFP 1.7 um column (ACE Excel) paired to a TSQ Quantiva Mass Spectrometer (ThermoScientific). Ten mM ammonium acetate buffer (eluent A) and 75% acetonitrile, 25% methanol with 10 mM ammonium acetate (eluent B) were used with a flow rate of 0.325 ml/min. For detection of bile acids, a gradient method was used starting at 26% eluent B for 5 min, and increasing to 98% eluent B by 25 min. A wash step was performed after each run using 100% methanol for 2 min, followed by equilibration in 100% eluent A for 5 min. Ionization of the samples was performed with the following settings: spray voltage, 3,500 V; vaporizer temperature, 350°C; sheath and auxiliary gas (nitrogen) pressure, 20 and 2 arbitrary units, respectively; ion transfer capillary temperature, 300°C; collision gas (argon) pressure, 1.5 mTorr; collision energy 10–55V; and ion polarity, negative. Selected reaction monitoring (SRM) was conducted using the characteristic precursor-to-product ion transition and retention times. Sample (1 uL) was injected into the LC-MS by autoinjector. Bile acid retention time was determined empirically using pure standards of each bile acid. Bile acid concentrations in samples were determined using standard curves for each bile acid.

### DNA Extraction

The majority of the samples were extracted with a protocol suitable for tough environmental samples because they contained a large amount of fibrous material. The protocol used a combination of bead beating and chemical lysis with a chloroform precipitation. Samples were lysed in 1 ml extraction buffer [100 mM Tris-HCl (pH 8.0), 100 mM EDTA disodium salt (pH 8.0), 100 mM sodium phosphate (pH 8.0), 1.5 M sodium chloride and 1% CTAB], 20 μl of proteinase K (10 mg/mL), and 25 μl of SDS (20%) using bead beating (0.25 g each of 0.1 mm and 0.5 mm glass beads) for 15 min on a horizontal vortex adaptor (MO BIO Laboratories, Carlsbad, CA) at full speed. Then, the samples were incubated at 60°C for 30 min with gentle end-over-end inversions by hand at the midpoint of 15 min; 750 μL of supernatant was collected in a new microcentrifuge tube after centrifugation at 6,000 × *g* for 10 min. DNA was separated from organic debris with a chloroform: isoamyl alcohol (24:1 vol/vol) extraction and precipitated overnight at −20°C using isopropanol. Samples were removed from the −20°C and warmed to 37°C to dissolve salt precipitates, and the DNA was pelleted at 15,000 × g for 30 min. Finally, the DNA pellet was washed twice with ice cold 70% ethanol and dissolved in 50 μL ultrapure water (NANOpure II™, Thermo Scientific, Waltham, MA, USA) water. The Recovery study samples were received at a later date and processed using methods developed for the NIH-Human Microbiome Project (34). DNA was extracted from feces using a MoBio Power Soil DNA extraction kit (MoBio, Carlsbad, CA) with added incubation at 65°C after blandk step as suggested for human samples.

### Library Preparation and Sequencing

Methods were used following the NIH-Human Microbiome Project (34). The V4 hypervariable region of 16S ribosomal RNA gene was amplified with barcoded primers [515f and 806r, (35)] in triplicate using the 5 PRIME HotMasterMix (VWR). Products were quantified with Quant-iT TM PicoGreen® dsDNA Assay Kit (Thermo Fisher) and samples were combined in equal amounts (~250 ng per sample) to be purified with the UltraClean PCR® Clean-Up Kit (MO BIO). Pooled amplicons were sequenced on the Illumina HiSeq 2,500 platform to generate 150bp single end reads.

Data from each study was processed individually to preserve their characteristics using Quantitative Insights Into Microbial Ecology (QIIME) software package version 1.9.1 (36). De-multiplexed and quality-controlled sequences were binned using open picking (37) with SUMACLUST (38, 39) into OTUs at 97% similarity using UCLUST against a Greengenes reference database using the pick_closed_reference_otus.py script (40, 41). Singletons and OTUs representing <0.005% total relative abundance were removed. Post-quality filtering and removing OTUs representing <0.005% of all OTUs were preformed to reduce the sparsity of the dataset, and a rarefied (42, 43) dataset was used in certain downstream analyses. The Oleic Blend Study had an average of 84,099 reads per sample and were rarified to 35,727 reads, which removed 1 of 30 samples. The Recovery Study had an average of 151,575 reads per sample and were rarified to 78,088 reads, which removed 2 of 96 samples. The Biliary Cholesterol Study had an average of 158,769 reads per sample and were rarified to 62,854 reads, which removed 0 out of the 32 samples. The Lipid Homeostasis Study had an average of 130,024 reads per sample and were rarified to 97,462 reads, which removed 1 of 12 samples.

### Statistical Analyses

Microbiome communities were visualized using unweighted UniFrac (44) with principal coordinates analysis (PCoA) using the phyloseq package (45). Differences among groups were tested using non-parametric multivariate analysis of variance (PERMANOVA) (46) by the adonis function (47). Alpha diversity was calculated using Shannon Diversity index, which takes into account richness and evenness, that is, if few taxa dominate the community or many taxa are evenly represented. Differential abundance was determined on non-rarefied data as suggested (42, 43) and normalized by size factors estimated by the median-of-ratios method using a negative binomial Wald Test that uses standard maximum likelihood estimates for Generalized Linear Model coefficients DESeq2 R package (48). *P*-values were corrected for multiple comparisons using Benjamini-Hochberg method and alpha was set to 0.01 using the. Analysis of variance (ANOVA) using Tukey *post-hoc* tests as a correction for multiple comparisons was used to detect significant differences in measured physiological traits. Bile acid data were analyzed with a two-way ANOVA accounting for the two treatments.

### Data Accessibility

The datasets analyzed for this study can be found in the NCBI Sequence Read Archive under accession number PRJNA701533 (Oleic Blend), PRJNA714188 (Recovery), PRJNA707361 (Lipid Homeostasis), and PRJNA713505 (Biliary Cholesterol).

## Results

### Overview of Studies

**Figure 1:**
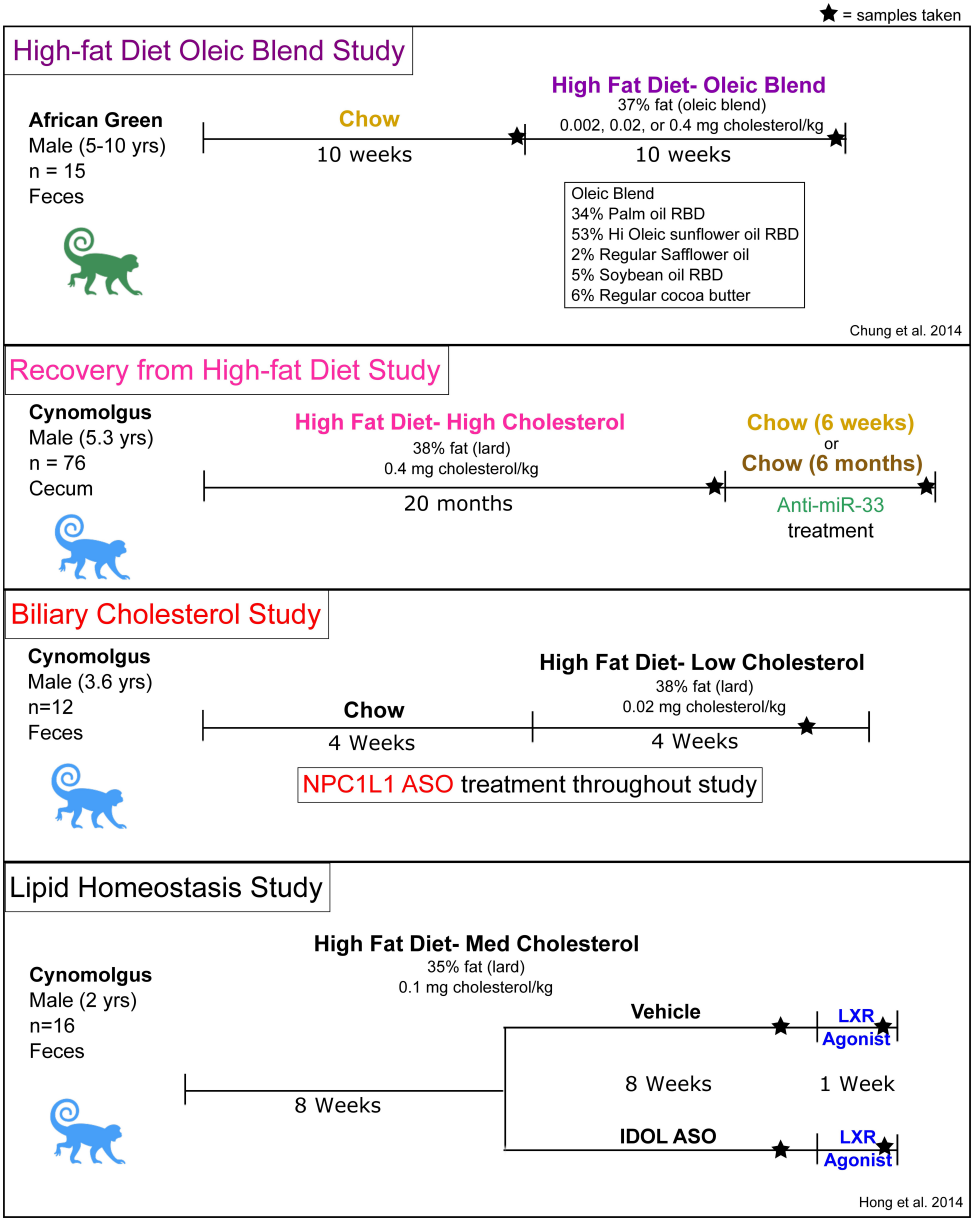
Overview of study designs, treatments, and diet compositions.

Feces and cecal samples were collected from previously completed NHP studies that utilized various treatments to influence plasma lipid levels (Figure 1). The studies were not designed to specifically test the effects of diet or pharmacological treatments on the microbiome, but their availability allowed us to investigate the relationship between the gut microbiome and host lipid metabolism. The high-fat diet Oleic Blend (HFD-OB) study was utilized to determine the effect of a monounsaturated fat rich diet in the context of three cholesterol levels in contrast to a chow diet. The Recovery study investigated changes that occur after being on a high-fat diet with high cholesterol (0.4% w/w or 1 mg/kcal) (HFD-HC) for a long period of time (20 months) and then during a chow recovery period with an anti-miR-33 or vehicle treatment. Targeting this microRNA was intended to increase HDL levels and improve cholesterol efflux (49), and consequently stimulate regression or stabilization of atherosclerotic lesions in the NHPs. Regardless of treatment type, switching animals from HFD-HC to chow diet resulted in a significant reduction in plasma total cholesterol (Supplementary Figure 1A). In contrast, anti-miR-33 compared to vehicle treatment significantly increased HDL cholesterol (Supplementary Figure 1B). The Biliary Cholesterol Study used a Niemann–Pick C1-Like 1 (NPC1L1) antisense oligonucleotide (ASO) throughout the duration of the study which we hypothesized would inhibit NPC1L1-mediated transport of biliary cholesterol into hepatocytes thereby decreasing hepatic and plasma cholesterol concentrations. The study design consisted of first a chow diet and then high-fat diet with low cholesterol (0.04% w/w or 0.1 mg/kcal) (HFD-LC). We found that NPC1L1 ASO treatment specifically decreased NPC1L1 gene expression in the liver resulting in modest reductions in hepatic and plasma but not biliary cholesterol (Supplementary Figure 2). Since bile acid concentration in gall bladder bile was also unchanged (data not shown), reducing hepatic NPC1L1 expression in liver does not appear to decrease the intracellular cholesterol pool to the point where bile acid synthesis and pool size would be diminished. The Lipid Homeostasis study used a high-fat diet with low cholesterol (0.05% w/w or 0.12 mg/kcal) (HFD-MC) for the duration of the study, but it also included ASOs for IDOL and LXR agonist. LXR agonist treatment of the cynomolgus monkeys increased LDL cholesterol due to increased hepatic IDOL and consequent degradation of LDL receptor protein (31). Treatment with the IDOL ASO resulted in partial knockdown of the expression of IDOL in the liver and significantly blunted the LXR agonist-mediated increase in LDL cholesterol (31).

Other details including monkey species and timescales were different between the studies. The high-fat diet oleic blend (HFD-OB) study was unique in that the primates were *Chlorocebus aethiops* (African green) and the high-fat diet was atypical. The remaining three studies used *Macaca fascicularis* (cynomolgus) primates with similar high-fat diet compositions and included treatments that influenced host gene expression related to cholesterol and lipid metabolism. The Recovery study was conducted over 2 years (Figure 1), but the other studies were completed within 9–20 weeks.

Each study contained a high-fat diet as part of the study design. The macronutrient levels were similar at 46% carbohydrates, 16–17% protein, and 37–38% fat (Table 1), but the lipid profile and components differed (Table 2). The high-fat diet oleic blend (HFD-OB) was 23% saturated fat, 64% monounsaturated fat, and 13% polyunsaturated fat, (Table 1) and was comprised of cocoa butter, palm, sunflower, safflower, and soybean oil (Table 2). There were also three levels of cholesterol: high (0.4 mg cholesterol/kcal), medium (0.02 mg cholesterol/kcal), and low (0.002 mg cholesterol/kcal). This oleic blend was created to determine the impact of a monounsaturated fat rich diet on atherosclerosis development in an African green monkey model. The fat source in the other diets was lard, and the composition was 46% saturated fat, 40% monounsaturated fat, and 19% polyunsaturated fat (Table 1). There were only two studies, HFD-OB and Recovery, able to compare high-fat diets to chow, and the chow diets did not have the same macronutrient levels or composition (Table 1).

**Table 2:** Diet composition of high-fat diets.

**Description**	**Ingredient**	**High-Fat Diet Composition (g/100g)**			
		**Oleic Blend**	**High Cholesterol**	**Medium Cholesterol**	**Low Cholesterol**
Carbohydrate	Dextrin	9.6	9.6	9.6	9.6
	Sucrose	10	10	10	10
	Wheat Flour, self-rising	35	35	35	35
Protein	Casein, USP	9	9	9	9
	Lactalbumin	5	–	–	–
	Fonterra Whey Protein Isolate-895	–	5	5	5
Lipid	ACHumko Oleic Blend^*^	16.4	–	–	–
	Lard	–	16.4	16.4	16.4
	Fish Oil (Omega Protein)	0.2	–	0.2	–
	Menhaden Oil (Omegapure)	–	0.2	–	0.2
	Crystalline Cholesterol	0, 0.08, 0.16	0.39	0.027	0.039
Fiber	Alphacel	7.3	6.5	7.3	6.5
Vitamins	Vitamin Mixture, Teklad ^*^ ^**^ ^***^	2.5	2.5	2.5	2.5
Mineral Salts	Hegsted Salt Mixture (Ca, P, Mg, Zn, Cu)	5	5	5	5
Plant Sterol	Beta-sitosterol (ICN)	0.0068	–	–	–
Antioxidant	Tenox 20A	0.008	–	0.008	–
Vitamin E	MTS-50 (NLT 50% total tocopherols, NMT 20% d-alpha tocopherol, oil)	0.012	–	–	–
Vitamin E	Vit E 5–67	0.004	–	–	–
	Calcium Carbonate	–	0.4	–	0.4

* *Complete Vitamin Mixture (BGSM formula) made by Teklad*.

** *Complete Vitamin Mix includes 0.0625 ml of D3 in Corn Oil for each 100 grams of diet ingredients*.

*(6.25 ml/10,000 gram batch of diet), to provide 2.5 IU of D3 per gram of diet*.

*** *All Calcium Phosphate Tribasic and Potassium Phosphate Dibasic was removed and replaced with Potassium Carbonate and Dextrin*.

*Water - 2000 ml/10,000 gm batch dry ingredients (3.36 Cal/gm wet diet)*.

*Feed monkeys 100−120 Calories per kg body weight per day + 10% waste*.

### Different High-Fat Diets With Opposite Diet Timing Similarly Affect the Microbiome

**Figure 2:**
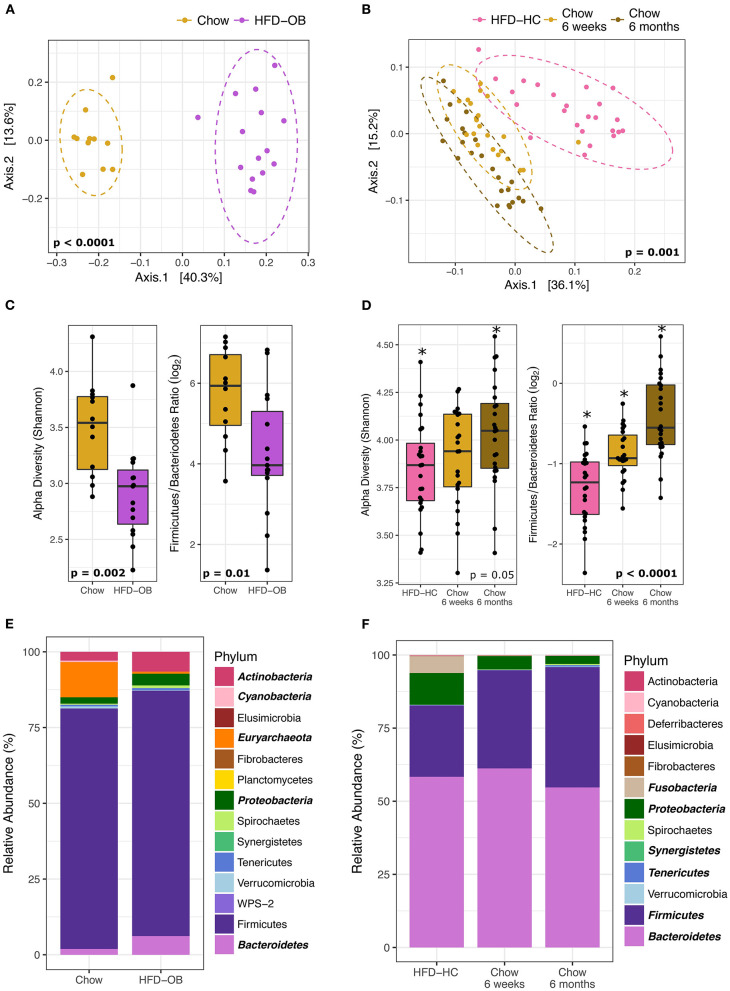
Microbiome changes between high-fat diets and chow are similar even with different diet composition and order. Left panels are the High-fat diet Oleic Blend study and right panels are the Recovery from a High-fat diet study. Ordinations **(A, B)** were plotted using principal coordinate analysis with weighted Unifrac distance, and groups were compared using non-parametric multivariate analysis of variance (PERMANOVA). Dashed ellipses represent 95% CI from the cluster centroid. Microbiome characteristics of alpha diversity (Shannon) and Firmicutes/Bacteroidetes Ratio (log_2_) were tested with Mann Whitney non-parametric *t*-test **(C)** or non-parametric Kruskal-Wallis ANOVA **(D)**. Differentially abundant phyla between diets were determined by DESeq2 using age and treatment as covariates and are bolded and italicized **(E)**. The two chow time points were combined for the RA study and time was added as a covariate in analyses **(F)**.

The contrasting effects between a high-fat and chow diet on the microbiome were more influential than the timing of the diet and the high-fat diet composition. The HFD-OB and Recovery studies both had high-fat and chow diets, but that is the only constant between them. All diets had different compositions, the time scale was weeks vs. months, and the diet orderings were opposite. The HFD-OB was a typical design where the chow diet was followed by a high-fat diet and each diet was fed for 10 weeks. The Recovery study started with 20 months of a high-fat diet, and then was followed by a chow diet for 6 weeks in trial 1 or 6 months in trial 2. Even with all of these differences, the effect of a high-fat diet similarly altered the community composition, visible in the principal coordinates analysis with Weighted UniFrac distance (Figures 2A,B). Significant clustering was determined by non-parametric multivariate analysis of variance (PERMANOVA) because it is sensitive to differences in group dispersion and location in the ordination, and therefore it is able to distinguish if groups are distinctly different. Both Recovery trials were included in the analysis because they were not significantly different (PERMANOVA, *P* = 0.135); however, the chow diet communities were different at 6 weeks vs. 6 months (PERMANOVA, *P* = 0.008) indicating a long-term response to coming off a high-fat diet. The effect of a high-fat vs. chow diet changed the microbiome community structure regardless of which diet was applied first, and the microbiome was still altered 6 months after switching from a high-fat to a chow diet.

Shifts in the microbiome due to diet were consistent. Alpha diversity (Shannon Index) and the Firmicutes/Bacteroidetes (log_2_) ratio were significantly decreased in the high-fat diets (Figures 2C,D) determined by Mann Whitney non-parametric *t*-test or non-parametric Kruskal-Wallis ANOVA. These values still continued to increase 6 months post high-fat diet indicating that the community had not yet reached equilibrium (Figure 2D). The long-term response of the microbiome to dietary changes has been noted in both mice (50) and humans (51). We may have expected the F/B ratio to increase on a high-fat diet because higher values have been associated with obesity (52), but the levels decreased in this instance. It is important to note that the animals did not become obese because they were fed controlled amounts of diet. We observed differences in the levels between studies, but this may be a result of different extraction methodologies. The Recovery study samples were processed at a later date using the protocols of the Human Microbiome Project while the HFD-OB study utilized a protocol consisting of a combination of bead sizes with chemical lysis that is suitable for tough environmental samples (53). It has been noted that extraction methods without multiple sizes of small beads underrepresent Firmicutes from a lack of lysis (personal communication, Zymo Inc.), and our data follows that pattern. Even with all of these differences, high-fat diets decreased diversity and F/B ratio regardless of diet composition and order indicating that these factors are outweighed by a macronutrient shift.

Phyla taxonomic level shifts were variable between the two studies. Differentially abundant phyla between diets were determined by DESeq2 using monkey age, treatment, and trial when applicable as covariates. The high-fat diet samples from the two Recovery study trials were combined and analyzed as one because there were no significant differences between them (PERMANOVA, *P* = 0.3). Increases in Bacteroidetes and Proteobacteria in the high-fat diets were the two consistent significant results (Figures 2E,F). Actinobacteria was greater in HFD-OB (Figure 2D) while Fusobacteria was greater in HFD-HC (Figure 2F). Decreases in phyla relative abundance on the high-fat diets were seen in Euryachaeota (11–0.7%) and Cyanobacteria (0.5–0.008%) in the Recovery study and Synergistetes, Tenericutes, and Firmicutes in the Oleic Blend study. All of these phyla, except for Firmicutes, represented <12% of the total relative abundance.

**Figure 3:**
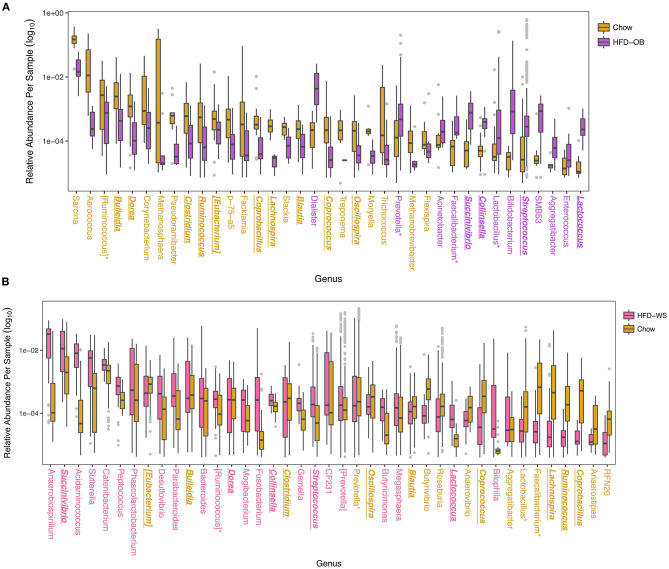
Differentially abundant taxa between high-fat and chow diets show overall consistency with a few disparate results. Significantly different OTUs are named by their genus level and were determined by DESeq2 using age and treatment, and time when applicable, as covariates. Colors for **(A)** High-fat Oleic Blend study and **(B)** Recovery from High-fat Diet study are ordered chronologically to represent which diet was received first. The HFD-OB contained three different levels of cholesterol and were combined for analysis because there were no significant differences between them. Colored taxa names refer to which treatment the abundance was higher. Taxa that have consistent trends between the two studies are bolded, italicized, and underlined, and taxa that have opposite trends are designated with an asterisk.

When analyzed at the OTU level, more taxa shifted in the same direction based on diet than not. Overall, the Recovery study had more significantly different OTUs at 329 vs. 195 OTUs in the Oleic Blend study. Thirty-three of these OTUs were common between the studies and 25 were consistent in which diet they were more abundant. The OTUs were averaged at the genus level to determine relevant taxa that were associated with the diets (Figure 3). In both studies, *Bulleidia, Clostridium, Ruminococcus, Eubacterium, Coprocacillus, Lachnospira, Blautia, Coprococcus*, and *Oscillospira* were greater in chow while *Succinivibrio, Collinsella, Streptococcus*, and *Lactococcus* were greater in high-fat diets. *Dorea, Prevotella, Faecalibacterium, Lactobacillus*, and *Aggregatibacter* had disparate results between the two studies. One difference between the studies is the proportion of significantly different genera that were greater in the high-fat diet where 54% (22/41) of the identified genera were in the Recovery high-fat diet and 15% (13/37) were in the Oleic Blend high-fat diet. The numbers followed the trend that whatever diet was received first had more significantly identified genera.

### Treatments That Did Not Affect the Microbiome

**Figure 4:**
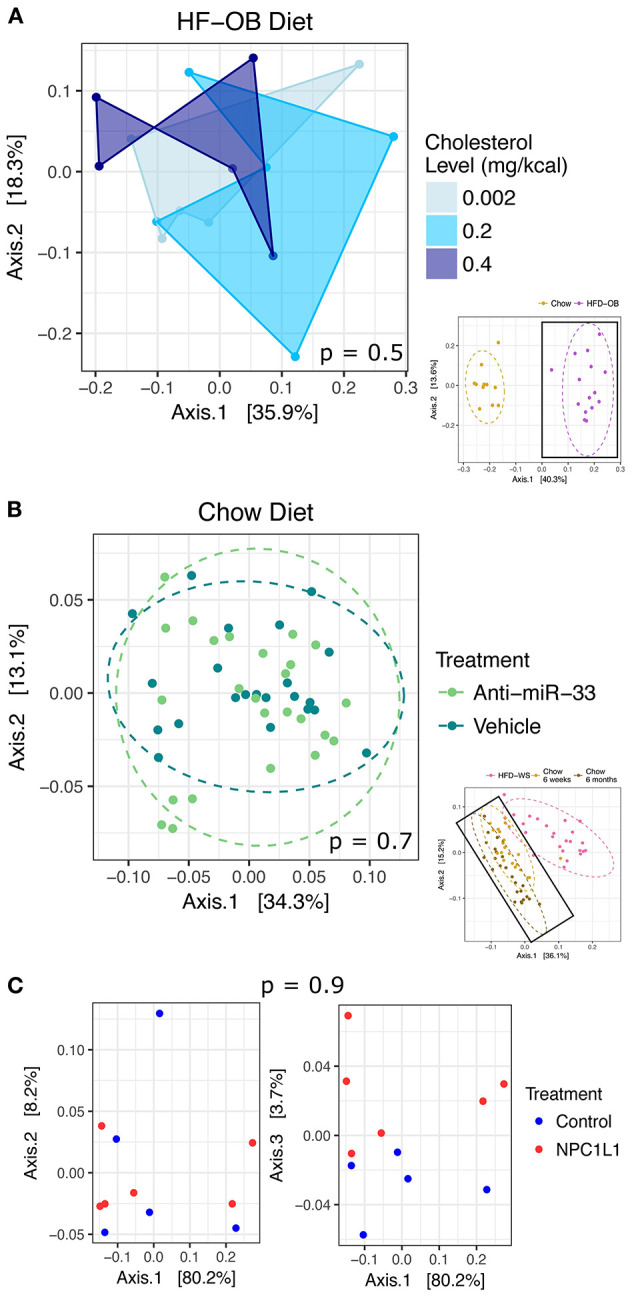
Treatments with no or minimal effect on the microbiome. Results were plotted using principal coordinate analysis with weighted Unifrac distance, and groups were compared using non-parametric multivariate analysis of variance (PERMANOVA). There was no effect in the **(A)** High-fat Oleic Blend study due to dietary cholesterol level and in the **(B)** Recovery from High-fat Diet study due to Anti-miR-33. There was a slight effect in the **(C)** Biliary Cholesterol study from NPC1L1 visible on Axis 3. Large ordinations represent samples enclosed in the bolded boxes of the thumbnail ordinations. Dashed ellipses represent 95% CI from the cluster centroid.

Various treatments were employed to alter plasma lipid levels and the microbiome was unresponsive to these. There was no distinct pattern of dietary cholesterol level associated with the microbiome community (Figure 4A) in the Oleic Blend study, but it did affect plasma total cholesterol (ANOVA, *P* = 0.001) and LDL (ANOVA, *P* = 0.0003) levels. The anti-miR-33 treatment was included during the chow period (6 weeks or 6 months) of the Recovery study, and it had no discernible effect on the microbiome (Figure 4B). The only plasma lipid that responded to the miR-33 ASO was HDL cholesterol, which significantly increased (*T*-test, *P* = 0.0002; Supplementary Figure 1). In the Biliary Cholesterol study, the NCP1L1 ASO was administered for the duration of the study. It modestly but significantly decreased plasma lipid and hepatic total cholesterol (Supplementary Figure 2), and showed a slight effect on the microbiome that is visible along Axis 3 in the PCA (Figure 4C). This axis represents only 3.7% of the variation, and the groups were not significantly different in the PCA plot (PERMANOVA, *P* = 0.9). The IDOL ASO of the Lipid Homeostasis Study was administered for 8 weeks and also had no influence (PERMANOVA, *P* = 0.141) on the microbiome. These results indicate that there is minimal or no relationship between host regulation of lipid levels through miR-33, NPC1L1, and IDOL and the microbiome.

### LXR Agonist Modifies the Microbiome

**Figure 5:**
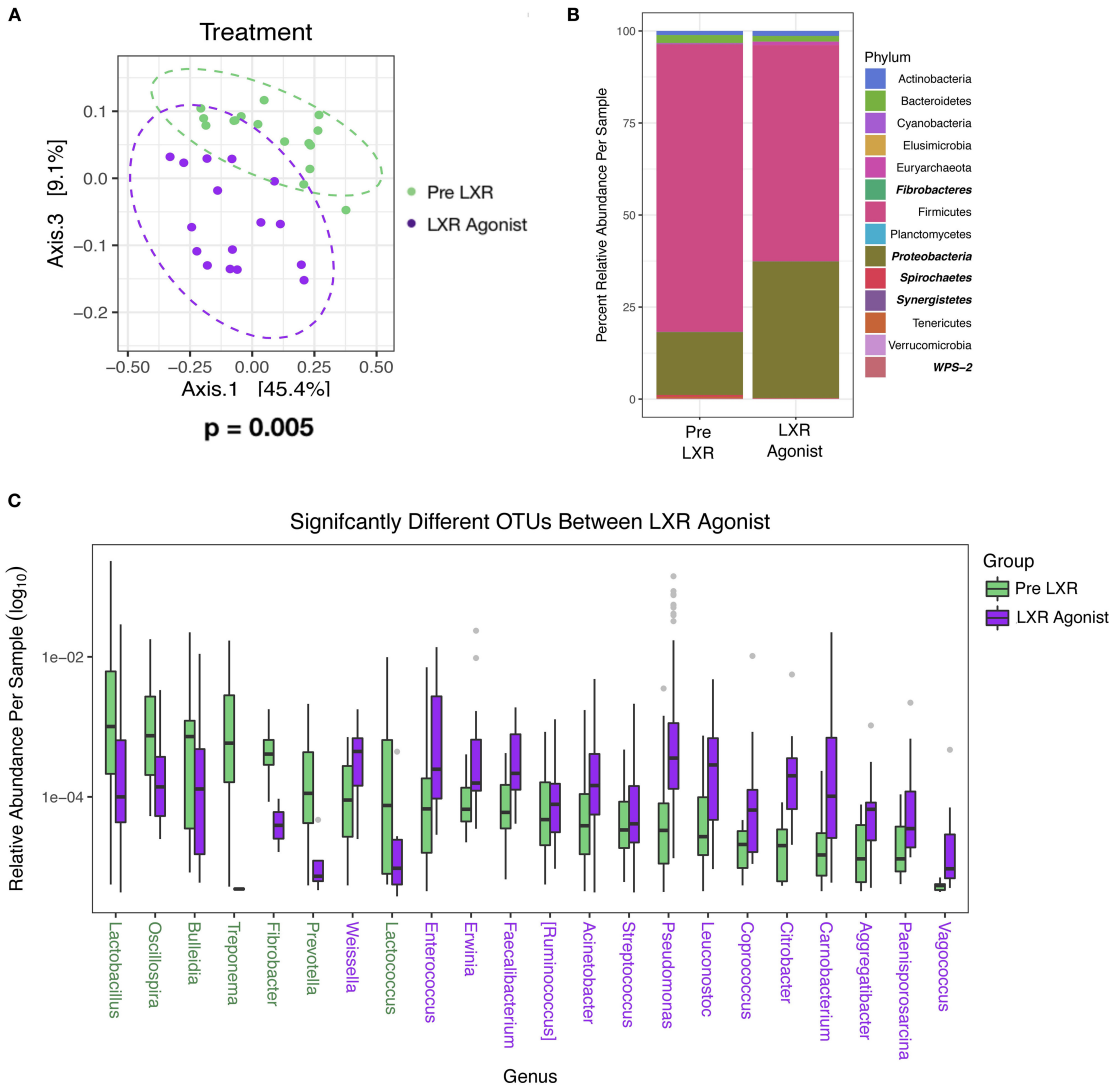
The Lipid Homeostasis Study used IDOL ASO and LXR Agonist as treatments, and only the LXR Agonist affected the microbiome. Samples were plotted using principal coordinate analysis with weighted Unifrac distance, and groups were compared using non-parametric multivariate analysis of variance (PERMANOVA) Dashed ellipses represent 95% CI from the cluster centroid. **(A)** Differentially abundant phyla between diets were determined by DESeq2 using age and treatment as covariates and are bolded and italicized **(B)**, and differentially abundant OTUs were described at the genus level **(C)**. Legend colors are listed in order corresponding to the ordering with the bars. If colors are missing, then the relative abundance values are too small to be visible. Colored taxa names refer to which treatment the abundance was higher.

One pharmacologic manipulation that altered the microbiome is the LXR agonist in the Lipid Homeostasis study. After 7 days of treatment, significant changes (PERMANOVA, *P* = 0.005) in the community were distinctly visible along Axis 3 in the PCA with Weighted UniFrac (Figure 5A). No significant differences were calculated by Mann Whitney non-parametric *t*-test in alpha diversity (Shannon; *P* = 0.42) and the Firmicutes/Bacteroidetes (log_2_) ratio (*P* = 0.70), but there were differences in specific taxa. Proteobacteria significantly increased (*P* < 0.0001) with the LXR agonist determined by DESeq2 using age and treatment as covariates, as did Synergistetes, but this phylum represented only 0.01% total relative abundance (Figure 5B). The phyla that significantly decreased with the LXR agonist were Fibrobacteres, Spirochaetes, and WPS-2, and these were also minimally abundant taxa representing 0.02, 0.16, and 0.04% total relative abundance, respectively (Figure 5B). When comparing at the OTU level, 106 OTUs were significantly different where 46 (representing 7 genera) were reduced and 61 (representing 15 genera) were increased with the LXR agonist. Fifty-four were described at the genus level, and OTUs within the same genus were averaged to determine the relative abundance (Figure 5C). Also, this treatment increased plasma total cholesterol (*t*-test, *P* = 0.0006) and LDL cholesterol (*t*-test, *P* = 0.002) levels (54), but not fecal cholesterol (*t*-test, *P* = 0.7), fecal coprostanol (*t*-test, *P* = 0.2), or fecal cholesterol excretion rate (*t*-test, *P* = 0.1) There is no known relationship between LXR and the microbiome, but LXR influences the conversion of cholesterol into bile acids by modulating a rate limiting step of cholesterol 7α-hydroxylase levels (55). Based on genomic profiling of bile salt hydrolase (BSH) genes in human gut microbiome communities (56), only 15% (7/29) of the OTUs that were increased in the Pre-LXR treatment may have had BSH activity while 46% (28/61) of the OTUs that were increased in the LXR agonist are in genera known to have BSH activity. In contrast, the Firmicutes phylum was decreased with the LXR agonist, which was the phylum that contained 59.73% of the surveyed BSHs (56). These observations cannot be tested statistically and would need further analyses to determine if there was a difference in BSH activity in response to the LXR agonist. The BSH containing genera of the OTUs increased in the pre-LXR treatment were *Ruminococcus, Lactobacillus, Streptococcus*, and *Treponema*, while the OTUs that increased in the LXR agonist were *Acinetobacter, Ruminococcus, Faecalibacterium, Pseudomonas, Streptococcus, Coprococcus*, and *Enterococcus*,. Only *Ruminococcus* and *Streptococcus* were present in both groups while the other genera were unique. These results suggest that bile acids could be indirectly responsible for a microbiome response to increased LXR activity.

### LXR Agonist Decreases Fecal Bile Acids

**Figure 6:**
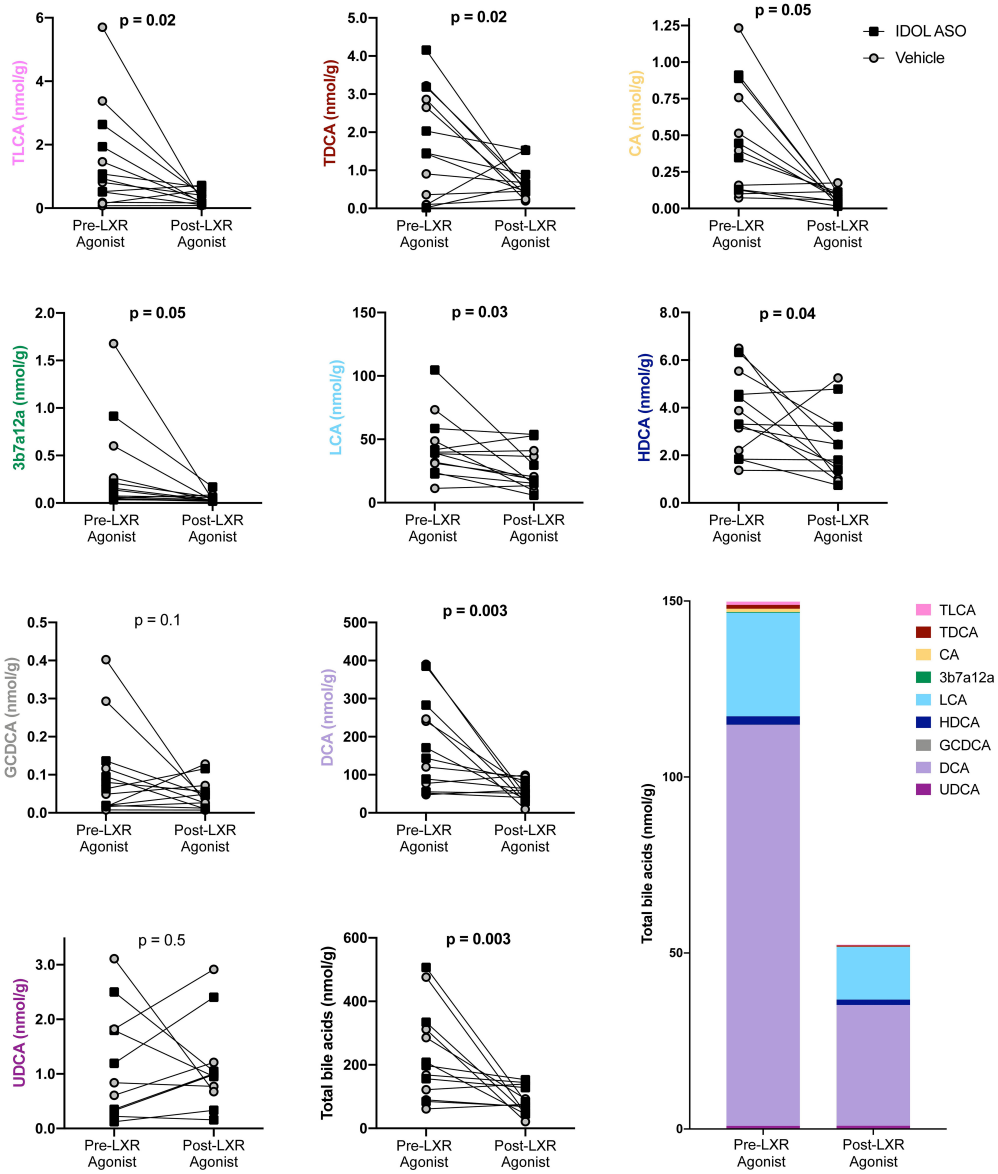
The LXR agonist decreased fecal bile acids. Data were analyzed with a two-way ANOVA for IDOL ASO and LXR Agonist treatments. There were no significant differences due to IDOL ASO, and the *p*-values plotted refer to the effect of the LXR treatment.

Bile acid levels in the feces were measured before and after the LXR agonist. A total of nine bile acids were detected. The primary bile acids cholic acid (CA) and glycochenodeoxycholic acid (CDCA) are synthesized from cholesterol by the liver. The secondary bile acids deoxycholic acid (DCA), lithocholic acid (LCA), hyodeoxycholic acid (HDCA), and ursodeoxycholic acid (UDCA) are a result of bacterial modifications that occur in the gut. Taurodeoxycholic acid (TDCA) and taurolithocholic acid (TLCA) are derivatives of the secondary bile acids DCA and LCA, respectively, that are absorbed by the host and then conjugated with taurine in the liver. The undefined bile acid 3b7a12a is an isomer of cholic acid. The most abundant bile acids were the bacterially derived DCA and LCA. Two genera were correlated to fecal bile acids using non-parametric Spearman correlation. *Pseudomonas* was negatively correlated to DCA (rho = −0.72, *P* = 0.006), CA (rho = −0.72, *P* = 0.006), total bile acids (rho = −0.68, *P* = 0.01), and 3b7a12a (rho = −0.63, *P* = 0.006), while *Ruminococcus* was positively correlated with TLCA (rho = 0.65, *P* = 0.03) and TDCA (rho = 0.63, *P* = 0.03). In general, the LXR agonist decreased fecal bile acid levels, and data was analyzed with a two-way ANOVA to account for the IDOL ASO and LXR agonist treatments (Figure 6). There was no significant effect of the IDOL ASO on any bile acids, but the LXR agonist significantly decreased total bile acids by two-thirds from 225 ± 140 (nmol/g) to 86 ± 43 (nmol/g) *(P* = 0.01). A significant decrease was also observed in all individual bile acids except GCDCA and UDCA (Figure 6). Bile acids in the gallbladder were measured, but all primates were on the LXR agonist treatment at study completion (Figure 1), therefore we were unable to compare pre- and post-LXR treatment. However, there were no significant differences due to the IDOL ASO on biliary bile acids. It is clear from these results that the LXR agonist decreases levels of bile acids in feces.

## Discussion

The compilation of these four studies refines the complex relationship between the microbiome and plasma lipid levels. The dietary influence on the microbiome is apparent and related to the macronutrient levels. The specific fat composition and cholesterol levels were not significant in this instance. Pharmacological treatments that influenced host lipid levels but not the microbiome included ASOs targeting miR-33, NPC1L1 and IDOL. This suggests that plasma lipid levels do not have a substantial direct effect on the microbiome whereas the microbiome can indirectly affect lipid levels (57, 58). One potential mediator between plasma lipid levels and the microbiome is bile acids. Our study shows that LXR agonist treatment shifted the microbiome. LXR affects bile acid levels and bile acids are toxic to bacteria and can shift the microbiome. This suggests that the indirect relationship between microbiome and plasma lipid levels is mediated by bile acids.

High-fat diets are known to shift gut microbe taxa levels and decrease diversity (59). Our results follow this pattern, even when the diet order is reversed, that is, where the high-fat diet is followed by the chow diet. Few studies have investigated this recovery of the microbiome after high-fat diets. When studied in mice, it took five times longer than the high-fat diet perturbation for the microbiome to revert back to a community resembling the normal chow (50). We observed that when placed on chow after a high-fat diet, communities remained different between 6 weeks and 6 months, and it is likely that the 6 month community was not yet at equilibrium. Mice were able to recover taxa suppressed by a low microbial accessible carbohydrate diet, which is inherent to a high-fat diet, but these were lost to the next generation (20). The time scale for the mouse study was weeks and it is unknown if 20 months of HFD consumption could cause eradication of the taxa that utilize the carbohydrate component of chow diets.

The Firmicutes/Bacteroidetes ratio has been utilized as a metric to describe the microbiome community and higher levels have been associated with high-fat diets (60) and obesity (52, 61, 62), but the pattern has not always held true in humans (63). We found that the ratio decreased on the high-fat diets, and this was opposite to trends previously observed in two different monkey studies. African green monkeys were fed a similar high-fat diet (protein- 18%, fat- 37%, carbohydrate- 45%, fiber-9%) and had an increased F/B ratio in contrast to decreases seen in humans (64). It is likely a factor other than fat level is driving the F/B ratio because higher levels were associated with wild populations in red-shanked doucs (*Pygathrix nemaeus*), with levels decreasing as the degree of captivity increased (65). Also, when comparing a western and Mediterranean diet that had nearly identical macronutrient levels in cynomolgus macaques (*Macaca fascicularis*), the F/B ratio was higher in the Western diet (66). The difference between the Western and Mediterranean diets was in saturated fat level (39 vs. 25%), monounsaturated fat (35 vs. 50%), omega 6:3 ratio (15:1 vs. 3:1), and fiber (9 vs. 13%) (66). This indicates that the ratio may be responding to specific nutrients that inherently change in chow compared to high-fat diets and it is not necessarily the difference in macronutrient levels.

Many of the identified taxa have been detected in other studies. A decrease in *Clostridium* and an increase in *Collinsella* were observed in both our high-fat diets and with African green monkeys on a similar macronutrient ratio Western diet (64). In the same study, an increase in *Cantenibactium* and *Desulfovibrio* was observed (64), but in our studies this only occurred in the Recovery one. *Prevotella* increased in their Western diet (64) similarly in our Recovery study but in contrast to the Oleic Blend study. In a study comparing Western diet to Mediterranean diet in cynomolgus macaques (66), a decrease in *Clostridium* and *Oscillospira* and an increase in *Coprococcus* was also observed in congruence with both our studies. However, an increase in *Ruminococcus* on their Western diet was opposite to the decrease in our studies, and a decrease in *Lactobacillus* and *Faecalibacterium* was only observed in the Recovery study. In addition, a meta-analysis using rodent and human data found that an increase in *Lactococcus* was the most reproducible result when comparing high-fat to chow diets (60), and this taxon was increased in both our high-fat diets.

The pharmacological ASO treatments used in this study were administered to alter plasma lipid levels of the host, and the negative results indicate that this regulation is not dependent on the microbiome. Anti-miR-33 treatment was deployed to aid recovery from a high-fat high-cholesterol diet because it increases HDL levels and reduces atherosclerotic plaque burden (49). The microbiome has been associated with plasma HDL levels (14), but we did not find any significant correlations, possibly because our study was underpowered. Transgenic expression of NPC1L1 in mouse liver results in significantly reduced biliary cholesterol and increased total plasma cholesterol (67); therefore it was hypothesized that ASO-mediated knockdown of hepatic NPC1L1 expression would increase biliary cholesterol and reduce plasma cholesterol. While hepatic knockdown of NPC1L1 was associated with modest but significant decreases in liver and plasma total cholesterol, the anticipated increase in biliary cholesterol was not found. A slight effect on the microbiome was observed with the NPC1L1 ASO, which could reflect differences in bile acid pool size or composition, which were not measured for this study. The IDOL ASO was used to prevent hyperlipidemia because it reduces IDOL expression and consequently increases LDL receptor, which is responsible for the clearance of LDL by the liver and other tissues (54). This treatment had no effect on the microbiome, and further supports that plasma lipid levels do not influence microbiome composition.

The one pharmacologic manipulation that did affect the microbiome was the LXR agonist. An effect was observed at 7 days, and this quick response was also seen in a different LXR study in cynomolgus monkeys (68). LXR activation has been shown to decrease cholesterol 7α-hydroxylase (CYP7A1), which is the rate limiting enzyme for conversion of cholesterol to primary bile acids (55) in human cells (69) and possibly African green monkeys (70). This would lead to a reduction of bile acid production and lowered lipid levels (68), which we observed. It is important to note that this is not the case in mice (68), which highlights the difference between species in relation to bile acids and lipid levels. In addition, the same LXR agonist was shown to increase inflammation in colonic cells through ATP-binding cassette transporter (ABCA1) (71). This transporter is also known as the cholesterol efflux regulatory protein (CERP) and influences cellular cholesterol levels. Since this treatment in our study affected fecal bile acid levels but not fecal cholesterol levels, we believe bile acids are related to the microbiome changes.

Bile acids are toxic to bacteria, and bile salt hydrolases are a conserved adaptation that removes taurine and glycine conjugations from bile acids making them more tolerable (72)–. Bacterial enzymes can also further modify bile acids to secondary bile acids through dehydroxylation, dehydrogenation, and epimerization reactions (26). These modified bile acids can then be reabsorbed and act as signaling molecules affecting multiple pathways of host metabolism (28). Two important receptors of these signals are nuclear farnesoid X receptor (FXR) and the G protein-coupled membrane receptor 5 (TGR5), and both of these are important to cardiovascular risk (29). These receptors have been discussed as a mechanistic route for bile acids affecting host lipid levels, but LXR has not been previously investigated (73). These results suggest that bile acids could shift the microbiome due to a manipulation of host lipid metabolism and that LXR may be another mechanistic route.

In summary, this compilation of non-human primate studies demonstrated that manipulations of host lipid levels through pharmacologic and dietary techniques may indirectly affect the microbiome through altered host bile acid regulation. High-fat diets compared to chow diets altered the microbiome in a consistent manner through decreased Shannon diversity and F/B ratio even when the fat sources were lard vs. a high oleic blend and the diets were applied in opposite orders. The relationship between host lipid levels and the microbiome has mostly been demonstrated through correlations and the mechanisms have remained unknown. We were unable to associate microbiome taxa with lipid levels due to power constraints, but we showed that pharmacological treatments of miR-33, NPC1L1, and IDOL ASO and dietary cholesterol did not affect the microbiome. The one treatment that did affect the microbiome was an LXR agonist and this may be through altered bile acid synthesis. Two genera, *Pseudomonas* and *Ruminococcus*, were correlated with various bile acids. These results indicate that the relationship between host lipid metabolism and the gut microbiome is likely indirectly regulated through the production of bile acids from cholesterol.

## Data Availability Statement

The datasets presented in this study can be found in online repositories. The names of the repository/repositories and accession number(s) can be found below: NCBI SRA repository, accession numbers: PRJNA701533 (Oleic Blend), PRJNA714188 (Recovery), PRJNA707361 (Lipid 392 Homeostasis), and PRJNA713505 (Biliary Cholesterol).

## Ethics Statement

The animal study was reviewed and approved by Institutional Animal Care and Use Committees of Wake Forest School of Medicine.

## Author Contributions

Monkey studies were designed by RT and samples were collected by LC, ZW, RL, and JL. JL generated, analyzed, and interpreted 16S data. LS conducted bile acid analysis and interpretation. CP provided statistical and analysis support. JL and AL wrote the manuscript. All authors contributed to the article and approved the submitted version.

## Conflict of Interest

JL and RT were employed by the company Novartis, and RL is employed by Ionis Pharmaceuticals. RT owns fifty shares of Ionis Pharmeceuticals stock. The remaining authors declare that the research was conducted in the absence of any commercial or financial relationships that could be construed as a potential conflict of interest.

## Publisher's Note

All claims expressed in this article are solely those of the authors and do not necessarily represent those of their affiliated organizations, or those of the publisher, the editors and the reviewers. Any product that may be evaluated in this article, or claim that may be made by its manufacturer, is not guaranteed or endorsed by the publisher.

## Supplementary Material

Supplementary Figure 1 Total and HDL cholesterol in NHPs from the Recovery from High-Fat Diet Study. Fasting plasma was isolated from male cynomolgus monkeys that were exclusively fed HFD-HC for 20 months (HFD-HC *n* = 24) or first fed HFD-HC for 20 months and then switched to a standard NHP “chow” diet and concomitantly treated with vehicle (6 weeks *n* = 13, 6 months *n* = 12) or miR-33 ASO (6 weeks *n* = 13, 6 months *n* = 12). Data for total cholesterol (A) was analyzed using Kruskal-Wallis test and Dunn's multiple comparisons test while that for HDL cholesterol (B) was analyzed using ordinary one-way ANOVA and Tukey's multiple comparisons test. Data not sharing a common letter differ significantly (*p* ≤ 0.02).

Supplementary Figure 2 Effects of NPC1L1 ASO on male cynomolgus monkeys in the Biliary Cholesterol study. **(A)** NPC1L1 mRNA in liver and small intestine. Data is graphed as relative expression (RE) to NPC1L1 in liver from vehicle treated animals. Liver data was analyzed using unpaired *t*-test and intestinal data was analyzed using Mann-Whitney test. **(B)** Cholesterol concentration of gall bladder bile. Data was analyzed using unpaired *t*-test. **(C)** Total and free cholesterol in liver. Data was analyzed using unpaired *t*-test. **(D–I**) Plasma cholesterol, LDL cholesterol and HDL cholesterol graphed as concentration (mg/dl) or percent change from baseline (Treatment Week 0). Data was analyzed using 2-way RM ANOVA, *p*-value for Time x Treatment.
