# Supplementary figures and images for: Dietary and Pharmacologic Manipulations of Host Lipids and Their Interaction With the Gut Microbiome in Non-human Primates
Source: Front Med (Lausanne). 2021 Aug 26;8:646710. doi: 10.3389/fmed.2021.646710 (PMC8426918; doi:10.3389/fmed.2021.646710)

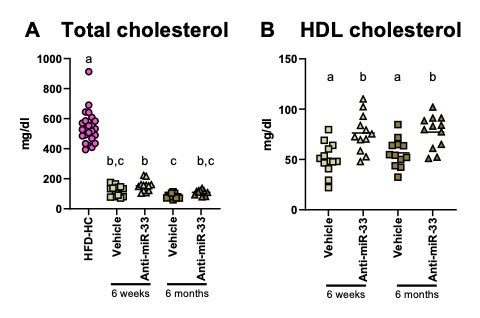

Supplement: Supplementary Figure 1 — Total and HDL cholesterol in NHPs from the Recovery from High-Fat Diet Study. Fasting plasma was isolated from male cynomolgus monkeys that were exclusively fed HFD-HC for 20 months (HFD-HC n = 24) or first fed HFD-HC for 20 months and then switched to a standard NHP “chow” diet and concomitantly treated with vehicle (6 weeks n = 13, 6 months n = 12) or miR-33 ASO (6 weeks n = 13, 6 months n = 12). Data for total cholesterol (A) was analyzed using Kruskal-Wallis test and Dunn's multiple comparisons test while that for HDL cholesterol (B) was analyzed using ordinary one-way ANOVA and Tukey's multiple comparisons test. Data not sharing a common letter differ significantly (p ≤ 0.02). [file Image_1.JPEG]

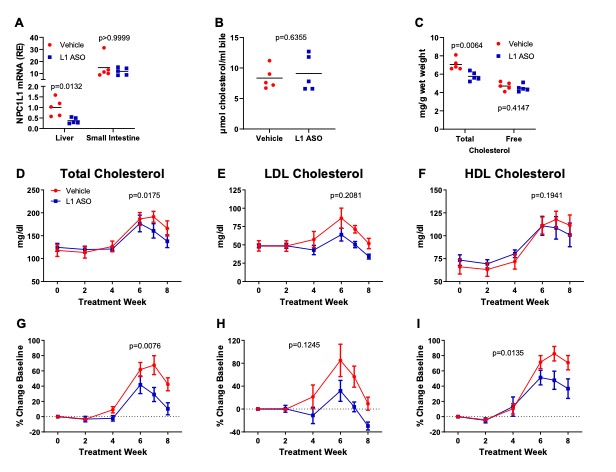

Supplement: Supplementary Figure 2 — Effects of NPC1L1 ASO on male cynomolgus monkeys in the Biliary Cholesterol study. (A) NPC1L1 mRNA in liver and small intestine. Data is graphed as relative expression (RE) to NPC1L1 in liver from vehicle treated animals. Liver data was analyzed using unpaired t-test and intestinal data was analyzed using Mann-Whitney test. (B) Cholesterol concentration of gall bladder bile. Data was analyzed using unpaired t-test. (C) Total and free cholesterol in liver. Data was analyzed using unpaired t-test. (D–I) Plasma cholesterol, LDL cholesterol and HDL cholesterol graphed as concentration (mg/dl) or percent change from baseline (Treatment Week 0). Data was analyzed using 2-way RM ANOVA, p-value for Time x Treatment. [file Image_2.JPEG]
